# Genome-wide expression datasets of anti-VEGF and dexamethasone treatment of angiogenesis in the rat cornea

**DOI:** 10.1038/sdata.2017.111

**Published:** 2017-08-15

**Authors:** Anthony Mukwaya, Pierfrancesco Mirabelli, Anton Lennikov, Maria Xeroudaki, Mira Schaupper, Beatrice Peebo, Neil Lagali

**Affiliations:** 1 Department of Ophthalmology, Institute for Clinical and Experimental Medicine, Faculty of Health Sciences, Linköping University, Linköping 58183, Sweden

**Keywords:** Rat, Microarray analysis, Angiogenesis, Inflammation, Animal disease models

## Abstract

Therapeutics against pathologic new blood vessel growth, particularly those targeting vascular endothelial growth factor (VEGF) are of enormous clinical interest. In the eye, where anti-VEGF agents are in widespread clinical use for treating retinal and corneal blindness, only partial or transient efficacy and resistance to anti-VEGF agents are among the major drawbacks. Conversely, corticosteroids have long been used in ophthalmology for their potency in suppressing inflammation and angiogenesis, but their broad biological activity can give rise to side effects such as glaucoma and cataract. To aid in the search for more targeted and effective anti-angiogenic therapies in the eye, we present here a dataset comparing gene expression changes in dexamethasone versus anti-*Vegfa* treatment of inflammation leading to angiogenesis in the rat cornea. Global gene expression analysis with GeneChip Rat 230 2.0 microarrays was conducted and the metadata submitted to Expression Omnibus repository. Here, we present a high-quality validated dataset enabling genome-wide comparison of genes differentially targeted by dexamethasone and anti-*Vegf* treatments, to identify potential alternative therapeutic targets for evaluation.

## Background & Summary

Pathological angiogenesis is a hallmark of many blinding diseases such as age-related macular degeneration, diabetic retinopathy, and corneal graft failure. In the eye, angiogenesis (or neovascularisation) is favoured by stimuli such as inflammation^1^, immunologic rejection, limbal stem cell deficiency, and hypoxia^2^. The choice of treatment for neovascularisation in the eye can be influenced by the stage of the neovascularisation process. While newly formed sprouts are usually leaky and susceptible to anti-VEGF treatment^3^, mature vessels require fewer angiogenic mediators^4^ necessitating other angioregressive therapies^5^. In the cornea in particular, therapeutics are used off-label to manage corneal angiogenesis, given the current lack of approved treatments specific for the cornea. Corticosteroids and/or nonsteroidal anti-inflammatory drugs (NSAIDs) are used to target neovascularisation^6,7^, however, steroids may promote infection^8,9^, glaucoma^10^, cataract^11^, and herpes simplex recurrence^12,13^ while NSAIDs are reported to cause corneal ulceration and melting^14^.

To date, targeting the VEGF family of pro-angiogenic factors is the most promising therapeutic approach as an alternative to steroid or NSAID use. Bevacizumab, a full-length recombinant humanized monoclonal antibody against VEGFA has been reported to treat corneal neovascularisation with some success^15,16^. Ranibizumab, a monoclonal antibody fragment also targeting VEGFA has similarly been shown to treat corneal neovascularisation^17^ and corneal graft rejection^18^. Compared to bevacizumab and ranibizumab, pegaptanib (a 28-base ribonucleic aptamer) is of limited efficacy, since it is specific to the VEGF-165 isoform^19^. The VEGF trap (aflibercept), a decoy receptor for all VEGFA isoforms, is the latest therapeutic approach being tested for use in the cornea^20^. In spite of the benefits of these anti-VEGF targeted therapies, some side effects of VEGF targeting have been documented, for example impaired corneal epithelialization and wound healing, a pathology attributed to the possible adverse effects of VEGF deficiency on nerve growth and regeneration^21,22^. In addition, anti-VEGF therapies are reported to be only partially effective^23,24^. Likewise, an antisense oligonucleotide against the insulin receptor substrate-1, aganirsen, was recently reported to reduce corneal neovascularisation only partially (by 26%) in patients with keratitis in a phase III study^19^. In a previous study we reported that topical administration of anti-*Vegf* led to a 14% reduction in neovessels compared to a 90% reduction with dexamethasone treatment in a rat corneal model of angiogenesis^25^.

In summary, there is need for new targeted therapies that mimic the potent effects of corticosteroids, but without the side effects attributed to their broad activity. A better understanding of specific factors suppressed by steroids but not by VEGF-A blockade is of potential importance for the development of more effective therapies. Here, we provide a detailed comparative dataset describing whole-genome differences in the activity of the corticosteroid dexamethasone, versus anti-VEGF treatment in a rat model of inflammatory corneal angiogenesis. GeneChip Rat 230 2.0 microarrays were used to monitor the global gene expression changes, and all microarray files including associated controls are described here, along with detailed information on the conditions of their generation and instructions for their re-use.

The dataset described here (Data Citation 1) can be a valuable resource for investigating genes and pathways to identify novel factors to target in order to improve the management of inflammation-associated neovascularisation in the cornea. The dataset described here has been thoroughly analysed in our related manuscript^26^, where it was shown that dexamethasone treatment led to a suppression of inflammatory chemokines and other genes within known signaling pathways like PI3K-Akt and focal adhesion, while notably anti-*Vegf* treatment did not have this suppressive activity. Furthermore, dexamethasone treatment led to an unexpected activation of the classical complement pathway^26^.

## Methods

The methods described here are an expansion of those described in our related work^26^.

### Suture model of inflammatory corneal angiogenesis

**Figure 1:**
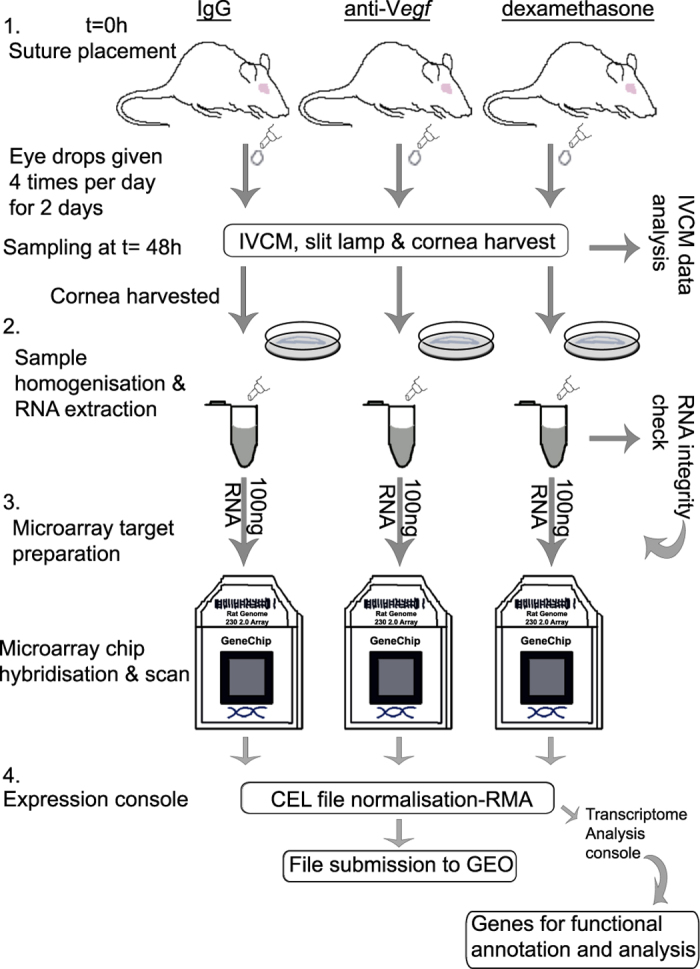
Schematic illustration of the experimental procedure that was followed. (1) sutures were placed intrastromally into the temporal cornea, and immediately followed by topical application of eye drops (IgG, anti-*Vegf* or dexamethasone). Eye drops were applied until the 48 h time point. At t=48 h, IVCM and slit lamp data was collected and used for phenotypic characterisation. (2) cornea tissue was harvested and used for RNA extraction, and RNA quality verified. (3) high quality RNA was used for target preparation for microarray hybridisation on to GeneChip Rat 230 2.0 microarray chips. The microarray chips were scanned and image files acquired. (4) CEL files were normalised using expression console software. The generated CHP together with the CEL files were submitted to Gene Expression Omnibus repository.

The model of suture induced cornea neovascularisation was used as previously described^1,25,27^. In summary, two nylon sutures were placed temporally to induce an inflammatory response that leads to new sprouting from limbal vessels after 2–3 days^27^. As a follow up of our previous findings^25^, here eye drops were administered topically immediately after suture placement i.e., during the pre-sprouting phase, but with high inflammation present in the cornea. At 48 h, *in vivo* confocal microscopy (IVCM) and slit lamp data was collected and corneal tissue was harvested for RNA processing. Global gene expression changes were assayed using microarrays. An illustration of the experimental design is shown in Fig. 1.

#### Treatment regime

Three groups of six rats each were treated with one of three topical treatments: IgG (Cat. No. 108-C, R&D Systems) at 20 μgml^−1^, anti-*Vegf* (Cat. No AF 564, a neutralizing rat-specific goat polyclonal pan-VEGFA antibody, R&D Systems, Minneaplois MN, USA) at 20 μgml^−1^ or dexamethasone (Opnol, Clean Chemical, Sweden AB, Borlänge, Sweden) at 1 mgml^−1^. The treatments were administered topically, 4 times daily, starting immediately following suture placement, and continuing up to 48 h (Fig. 1). Additionally, a group of four non-sutured and non-treated corneas was harvested to serve as the control.

#### Sample collection and RNA extraction

After 48 h of topical treatment, animals were euthanized and a standardized region of tissue between the sutures and limbus which indicated a strong inflammatory response by *in vivo* confocal microscopy^26^ was carefully dissected out. The tissue was harvested from all groups (i.e., IgG, anti-*Vegf*, dexamethasone and non-sutured/non-treated control), six animals per treatment group and then immediately immersed in RNA later (Qiagen) and temporarily stored at 4 °C. Total RNA was then extracted from all the six samples using Qiagen mini RNA preparation kit (Qiagen). RNA concentration was then determined using a NanoDrop spectrophotometer (Thermo Scientific), and RNA quality was determined using the Agilent bioanalyzer 2100 (Agilent Technologies). RNA integrity number (RIN)≥7 was set as the cut-off for sample inclusion for downstream processing for microarray analysis. Of the six RNA samples extracted per treatment group, four RNA samples per treatment group (with highest RIN) were selected for downstream processing.

#### Microarray target preparation

GeneChip Rat 230 2.0 microarrays were used for global gene expression analysis. Four microarray chips were used per experimental group, including non-sutured and non-treated control corneas. Each microarray chip represented a single corneal sample (16 microarrays in total) i.e., no pooling of cornea tissue was performed. As per the protocol (Manual Target Preparation for GeneChip 3′ Expression Arrays-Affymetrix), a total of 100 ng of high quality RNA per sample was mixed with poly-A spike-in Controls, and the mixture was used for first-strand synthesis to yield single-stranded cDNA with the T7 promoter sequence at the 5′ end. Next, using a DNA polymerase and RNase H, the single-stranded cDNA was converted to double-stranded cDNA to serve as a template for *in vitro* transcription using T7 RNA polymerase. This resulted in the synthesis of labelled complementary RNA (cRNA). The labelled cRNA was then purified, and then both the yield and size distribution was verified using a NanoDrop spectrophotometer. The labelled cRNA was then fragmented and used for the preparation of the array hybridisation master mix. Samples were hybridised to the microarray chips at 60 rpm for 16 h, at 45 °C in GeneChip Hybridization Oven 645 (Affymetrix.inc). The microarray chips were washed and stained in a GeneChip Fluidics Station 450 (Affymetrix.inc.) and then scanned in a GeneChip Scanner 3000 7 G (Affymetrix.inc.).

## Data Records

Following data acquisition, the CEL files were normalised, and CHP files were generated using Expression Console Software (Affymetrix. Inc). The CEL and CHP files were submitted to Gene Expression Omnibus repository (Data Citation 1). The samples used in the study are summarised in Table 1.

## Technical Validation

Following suture placement, randomisation was performed in assigning the rats to different treatment groups. For the purpose of statistical inference, a total of four rats was used per treatment to correspond to four microarray chips per treatment group. ANOVA was used to compare effects of the given treatments across treatment groups, with Tukey multiple comparison test used to isolate pairwise differences.

Efficacy of the anti-*Vegf* treatment was verified by Western blot analysis prior to the beginning of the study to confirm the expected effect on the gene target^26^. Suture placement was performed by the same experienced surgeon for all treatment groups, to reduce the chances of technical variation. In addition, a similar phenotypic appearance within each group was confirmed by slit lamp biomicroscopy and *in vivo* confocal microscopy^26^.

### RNA quality check

**Figure 2:**
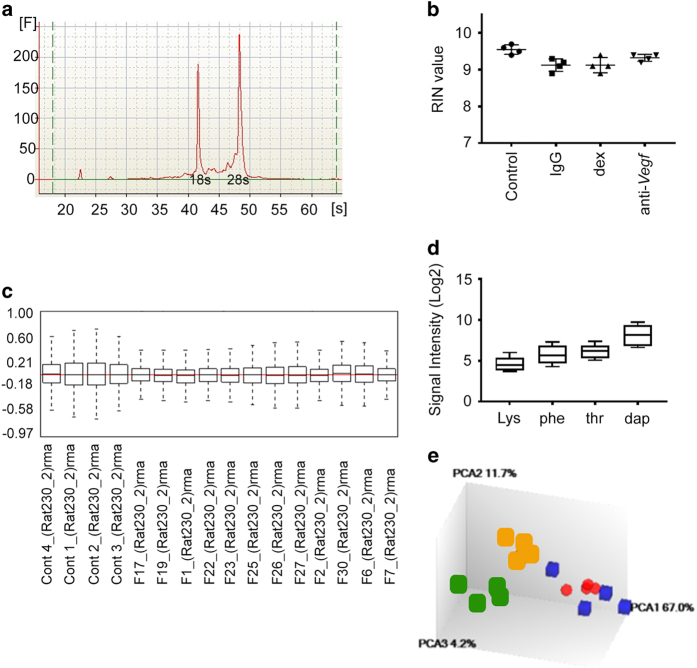
RNA and microarray data quality check. Following Bioanalyser RNA quality check, (**a**) is an electrophoregram showing RNA sample quality. The distinct peaks in (**a**) represent the 18 and 28 s rRNA. (**b**) are the obtained RIN values across treatments. (**c**) is a display of the Relative Log Expression Signal-RMA, while (**d**) are Boxplot of four of Affymetrix Spike-In controls, lys-M, phe-M, thr-M and dap-M illustrating the range of signal (log2) over the experiment as well as quality control of the arrays technical run. (**e**) is the PCA-Probe Cell Intensity Data.

The quality of the RNA was checked prior to downstream analysis, using the Agilent Bioanalyser 2100 (Agilent Technologies), using the 2100 expert_Eukaryote Total RNA Nano settings. The analysis showed clear, defined 28 and 18 s rRNA peaks, an indication of high quality RNA. Moreover, low noise between the peaks, and minimal low molecular weight noise was detected. In Fig. 2a an elctrophoregram is presented illustrating the peaks for the 18 and 28 s rRNA, and in Fig. 2b the sample RIN values are given.

### Quality control of microarray data

The raw CEL files were converted into expression measures, background-corrected, and data-normalized using the RMA method^28^, with the help of Expression Console Software (Affymetrix. Inc). In Fig. 2c the relative log expression Signal-RMA is given. In Fig. 2d are boxplots representing four of the Affymetrix Spike-In controls, lys-M, phe-M, thr-M and dap-M illustrating the range of signal (log2) over the experiment. In Fig. 2e is the PCA-Probe Cell Intensity Data. The PCA1 (67.0%) represents anti-*Vegf* and IgG treated groups in blue and red colours, respectively. PCA2 (11.7%) represents dexamethasone treated group in the orange colour, while PCA3 (4.2%) displays the control samples in green colour. Note the partial overlap of anti-*Vegf* and IgG groups and clear separation from the dexamethasone treated corneas.

**Figure 3:**
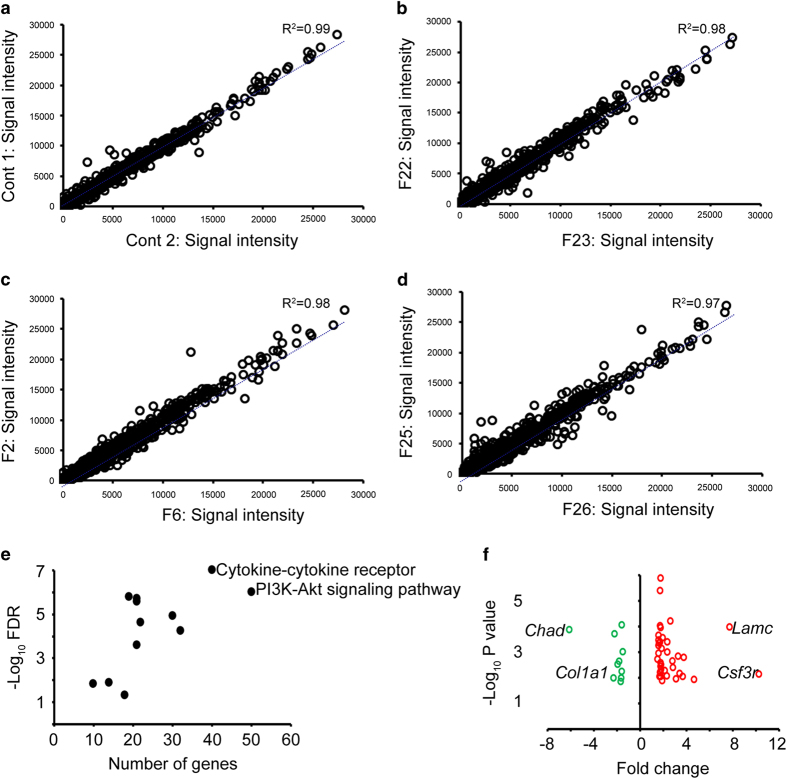
A correlation analysis within treatment group. (**a**–**d**) are signal intensity values correlated between control, IgG, anti-*Vegf* and dexamethasone treated samples respectively. (**e**) is an example of pathway enrichment analysis and (**f**) is a display of the genes involved in a selected pathway (PI3K-Akt signalling pathway).

The CHP files were used for gene expression analysis using Transcriptome Analysis Console (TAC) Software (Affymetrix, Inc.). Using the obtained linearized signal intensity values from the TAC analysis, a correlation analysis between samples within the same treatment groups was performed. The resulting scatter plots with their corresponding correlation coefficient (R^2^) are presented in Fig. 3a–d. There was strong correlation within samples of the same treatment group. The linearized values can then be log transformed and used for functional annotation analysis. As an example, STRING^29^ pathway enrichment analysis was performed using the differentially expressed genes in the dexamethasone treated group. Selected pathways from this analysis are presented in Fig. 3e, and the genes involved in one of the pathways (PI3K-Akt signalling pathway) are represented as an example in Fig. 3f.

### Gene fold change by qPCR

**Figure 4:**
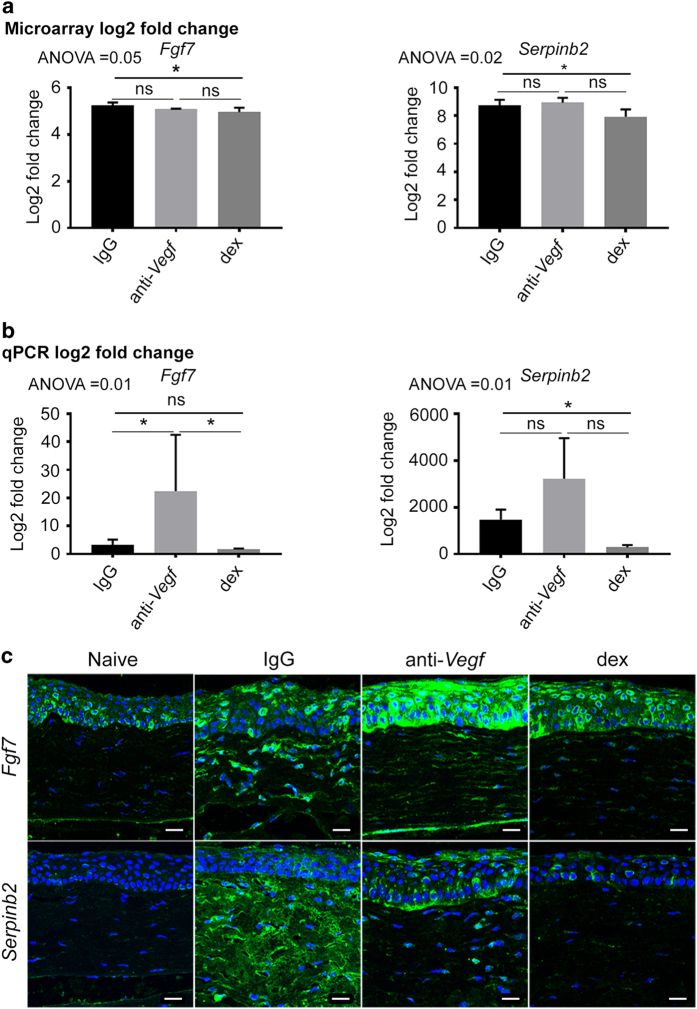
Gene fold change, and localisation of target protein expression in cornea sections. (**a**) is the gene fold change by microarray. (**b**) is the gene fold change by qPCR. *n*=4 and 3 corneas for (**a**,**b**), respectively. ANOVA followed by Tukey’s multiple comparisons test was used to isolate pairwise differences, with alpha <0.05. In both (**a**,**b**), asterisks represent adjusted *P*<0.05 and the error bars represent standard deviation. ns, not significant. The data in (**a**,**b**) was analysed using GraphPad Prism 5 (GraphPad Software, Inc. CA 92037 USA). (**c**) is the localisation of the corresponding protein expression in cornea cross sections stained by immunohistochemistry. White scale bars represent 20 μm.

To validate microarray^30^ gene fold change (Fig. 4a), here we used a separate set of rats (3 per treatment group) for qPCR analysis. Rats were sutured and treated identically as for microarray samples. At the 48 h time point, animals were sacrificed and cornea tissue was harvested and used for RNA extraction, without pooling. Following RNA extraction, cDNA was synthesised using superscript cDNA synthesis kit (Invitrogen), and used for qPCR analysis using TaqMan primers. Genes *Fgf7* and *Serpinb2* were assayed as exemplary genes, with results shown in Fig. 4b.

### Localisation of translated genes

Cornea samples from the respective treatment groups were harvested and fixed in paraformaldehyde for 24 h, and then stabilised in paraffin blocks. Then 5 μm cross-sections of the tissue were made. Prior to staining, the sections were deparaffinised and rehydrated. The sections were probed with primary antibody for *Fgf7* and *Serpinb2*, and detected with fluorescently (Alexa 488) labelled secondary antibody Fig. 4c. Images were acquired using a laser scanning confocal fluorescent microscope (Zeiss LSM700).

## Usage Notes

The raw CEL files can be normalised using Expression console, and gene fold change can be obtained using Transcriptome Analysis Console. Both software packages can be freely accessed from Affymetrix (Affymetrix Inc).

Transcriptome Analysis Console (Affymetrix Inc) can be used for pathway enrichment analysis, where both pathway *P*-value and significance can be obtained. The genes involved in a given pathway can also be mapped within a pathway of interest.

Also using other publicly available software for bioinformatics analysis such as STRING^29^, Venny^31^, DAVID^32^, PANTHER^33^, etc., and those commercially available like Ingenuity pathway analysis (IPA-Qiagen) (http://www.ingenuity.com/), the differentially expressed genes can be used to deduce pathway and biological processes of interest, and to further isolate the genes involved in them.

In combination with our recently published Data Descriptor^34^, genes important for endogenous restoration of corneal avascularity during either an angiogenic sprouting or regression phase can be compared to their behaviour observed with either dexamethasone or anti-*Vegf* treatment in the current dataset, to gain an understating of how these treatments modulate these factors.

## Additional Information

**How to cite this article:** Mukwaya, A. *et al.* Genome-wide expression datasets of anti-VEGF and dexamethasone treatment of angiogenesis in the rat cornea. *Sci. Data* 4:170111 doi: 10.1038/sdata.2017.111 (2017).

**Publisher’s note:** Springer Nature remains neutral with regard to jurisdictional claims in published maps and institutional affiliations.

## Supplementary Material



## Figures and Tables

**Table 1:** Dataset and sample description across treatment groups.

**GSM-ID**	**ID**	**Organism**	**Age (Weeks)**	**Description/ Treatment**	**Sample type and source**	**Technology**
GSM2327895	F1	rattus norvegicus albino	12	anti-*Vegf*_F1	RNA-Cornea	Microarray
GSM2327896	F2	rattus norvegicus albino	13	anti-*Vegf*_F2	RNA-Cornea	Microarray
GSM2327897	F6	rattus norvegicus albino	12	anti-*Vegf*_F6	RNA-Cornea	Microarray
GSM2327898	F7	rattus norvegicus albino	13	anti-*Vegf*_F7	RNA-Cornea	Microarray
GSM2327899	F17	rattus norvegicus albino	12	IgG_F17	RNA-Cornea	Microarray
GSM2327900	F19	rattus norvegicus albino	13	IgG_F19	RNA-Cornea	Microarray
GSM2327901	F22	rattus norvegicus albino	13	IgG_F22	RNA-Cornea	Microarray
GSM2327902	F23	rattus norvegicus albino	12	IgG_F23	RNA-Cornea	Microarray
GSM2327903	F25	rattus norvegicus albino	13	dexamethasone_F25	RNA-Cornea	Microarray
GSM2327904	F26	rattus norvegicus albino	13	dexamethasone_F26	RNA-Cornea	Microarray
GSM2327905	F27	rattus norvegicus albino	12	dexamethasone_F27	RNA-Cornea	Microarray
GSM2327906	F30	rattus norvegicus albino	12	dexamethasone_F30	RNA-Cornea	Microarray
GSM2327907	Cont 1	rattus norvegicus albino	13	Control 1	RNA-Cornea	Microarray
GSM2327908	Cont 2	rattus norvegicus albino	13	Control 2	RNA-Cornea	Microarray
GSM2327909	Cont 3	rattus norvegicus albino	12	Control 3	RNA-Cornea	Microarray
GSM2327910	Cont 4	rattus norvegicus albino	12	Control 4	RNA-Cornea	Microarray
